# Evolution of Spinal Cord Swelling in Acute Traumatic Spinal Cord Injury

**DOI:** 10.1089/neur.2025.0005

**Published:** 2025-02-12

**Authors:** Hasan Asif, Ravindran Visagan, Ellaine Boseta, Argyro Zoumprouli, Marios C. Papadopoulos, Samira Saadoun

**Affiliations:** ^1^ Academic Neurosurgery Unit, Neuroscience and Cell Biology Research Institute, St. George’s, University of London, London, UK.; ^2^ Neuro-Intensive Care, St. George’s Hospital NHS Foundation Trust, London, UK.; ^3^ Neuro-Anaesthesia, St. George’s Hospital NHS Foundation Trust, London, UK.

**Keywords:** critical care, edema, Monro–Kellie, spinal cord injury

## Abstract

We hypothesized that the Monro–Kellie doctrine, a key principle in traumatic brain injury (TBI), also applies in traumatic spinal cord injury (TSCI). By analyzing 9986 h of intraspinal pressure (ISP) monitoring data from 79 TSCI patients, we show that concepts developed to quantify compensatory reserve in TBI may be analogously defined in TSCI, termed ISP pulse amplitude (sAMP), spinal compensatory reserve index (sRAP), and ISP waveform shape. As ISP increases beyond 15 mmHg, compensatory reserve becomes impaired (sAMP rises and sRAP becomes positive). As ISP increases beyond 20 mmHg, the morphology of the ISP waveform changes from three peaks (P1, P2, P3) with P1 dominant, to three peaks with P2 dominant, to a rounded signal. Key differences in TSCI, compared with TBI, are no plateau ISP waves, and no critical ISP beyond which sAMP decreases and sRAP becomes negative. Four factors were associated with increased spinal cord swelling or reduced spinal cord compliance: thoracic level of injury, no laminectomy, delayed surgery, and more severe injury. We also hypothesized that, as in TBI, the spinal cord maximally swells a few days after injury. Serial ultrasound scans of the injured spinal cords in 9 patients and plots of change from baseline in ISP, sAMP, and sRAP versus time in 79 patients revealed delayed maximal cord swelling within 5 days of surgery. We conclude that the spinal Monro–Kellie concept allows the spinal compensatory reserve to be quantified. Our data show that spinal compensatory reserve becomes exhausted as ISP increases above 15–20 mmHg and that there is delayed cord swelling after injury, which implies that adequate cord decompression confirmed during surgery by ultrasound may not persist postoperatively.

## Introduction

To optimize the management of patients with traumatic spinal cord injuries (TSCI) in the neurointensive care unit (NICU), we monitor intraspinal pressure (ISP) and spinal cord perfusion pressure (SCPP), by placing a subarachnoid pressure probe at the injury site.^1^ The ISP signal is similar to intracranial pressure (ICP) with three peaks (P1, P2, and P3) and comparable cardiorespiratory frequency components.^1,2^ ISP and SCPP monitoring in TSCI are analogous to ICP and cerebral perfusion pressure (CPP) monitoring in traumatic brain injury (TBI).^3^

We hypothesized that the Monro–Kellie doctrine,^4^ a key principle in TBI, also applies in TSCI. The spinal Monro–Kellie is as follows: as the injured cord swells, it displaces the surrounding cerebrospinal fluid (CSF), which prevents a rise in ISP. With further cord swelling, there is no CSF left around the injured cord; thus, the cord becomes compressed against the theca, and ISP rises. The spinal Monro–Kellie hypothesis implies that the exponential pressure–volume relationship in the brain^5^ may also apply to the spinal cord. This leads to three testable predictions as follows: First, by analogy with ICP pulse amplitude termed AMP,^5,6^ as ISP increases, ISP AMP (spinal AMP, sAMP) is initially unchanged, then increases. Second, compensatory reserve, quantified in TBI using RAP,^4,7,8^ may be analogously quantified in TSCI using spinal RAP (sRAP) defined as the running correlation coefficient (R) between sAMP (A) and mean ISP (P). With intact compensatory reserve, sRAP ≈0; as compensatory reserve becomes exhausted, sRAP ≈1. Third, as with ICP, P2 in ISP is compliance. By analogy with ICP,^5,9^ at low ISP, P1 dominates, but as compensatory reserve is exhausted, P2 dominates, and the ISP waveform ultimately becomes rounded with P2, the only detectable peak.

We also hypothesized that the rise in ICP observed a few days after TBI as brain edema increases and contusions blossom^10,11^ also applies in TSCI, that is, the ISP peaks a few days after the TSCI. Delayed cord swelling implies that adequate cord decompression, shown by ultrasound scanning (USS) during surgery, may not persist. To address this hypothesis, we defined the evolution of spinal cord swelling after TSCI by ISP monitoring and serial USS.

## Materials and Methods

### Institutional research board approvals

Patients were recruited as part of the Injured Spinal Cord Pressure Evaluation (ISCoPE) study at St George’s Hospital. Approvals for ISCoPE were granted from the St George’s, University of London Joint Research and Enterprise Service and the National Research Ethics Service London-St Giles Committee (10/H0807/23). ISCoPE is registered at www.clinicaltrials.gov and www.ichgcp.net as NCT02721615. The study was conducted in accordance with the ethical standards, as agreed in the 1964 Declaration of Helsinki and its subsequent amendments. Informed consent was obtained from all participants or family members.

### Patients

All TSCI patients recruited into ISCoPE between October 2010 and March 2024 were included. The inclusion criteria were as follows: (1) severe TSCI (American spinal injury association Impairment Scale, AIS, grade A–C), (2) age 18–75 years, and (3) surgery within 72 h of TSCI. The exclusion criteria were as follows: (1) major comorbidities—active malignancy, severe chronic kidney disease, liver disease with cirrhosis and portal hypertension, congestive heart failure, recent myocardial infarction or pulmonary embolus, stroke with hemiplegia, (2) other central nervous system disease-demyelinating, vascular, and/or space-occupying lesions with preexisting neurological deficit, (3) penetrating TSCI, and (4) patients with lumbar drains *in situ*.

### Neurosurgical management

Patients were admitted to the neurosurgical unit at St. George’s Hospital and underwent International Standards for Neurological Classification of Spinal Cord Injury assessments by a trained neurosurgical resident, which were repeated at follow-up. Surgery was performed by an appropriately trained neurosurgeon. The type of surgery was based on surgeon preference that included a posterior approach with laminectomy or laminotomy, with or without lateral mass (cervical) or pedicle (thoracic) screw and rod fixation (Stryker Oasys for cervical, Stryker Xia for thoracic; Stryker, Newbury, Berkshire, England). After laminectomy, a pressure probe (Codman Microsensor Transducer; Depuy Synthes, Leeds, United Kingdom) was inserted under the operating microscope. The pressure probe was placed between the spinal cord and the arachnoid at the site of maximal cord swelling, tunneled out, and secured to the skin with nonabsorbable sutures. The skin was closed with nylon suture. Patients had computed tomography (CT) and magnetic resonance imaging (MRI) of the spine before surgery, CT within 48 h and MRI at 1–2 weeks postoperatively.

### NICU management

Postoperatively, patients were admitted to the NICU and were reviewed daily by the NICU and neurosurgical teams. Ventilation was supported as appropriate, including timely extubation or early tracheostomy. A passive wound bed drain was kept for a duration of 7 days. Patients were turned every 2 h in bed to avoid decubitus ulcers. Thromboembolic deterrent stockings with intermittent pneumatic compression devices were applied and prophylactic low-molecular-weight heparin was commenced at 24 h after surgery to reduce the risk of venous thromboembolism. Blood pressure was monitored invasively using a radial arterial line. Mean arterial pressure (MAP) management was at the discretion of the NICU team, in general maintaining MAP ≥80 mmHg, using noradrenaline (norepinephrine) if needed. The NICU team was blinded to the ISP reading and no interventions were given to adjust ISP or SCPP. The pressure probe was removed on the NICU, and the exit sites were sutured.

### Ultrasound scanning

USS was performed intraoperatively and postoperatively from August 2022.

#### Intraoperative

After laminectomy and before inserting the pressure probe, the surgical cavity was filled with warm saline. Spinal cord USS was performed with an Aloka ProSound Alpha 7 machine (Hitachi, Tokyo, Japan) using an intraoperative convex probe (UST-9120, Hitachi) at 4–10 MHz. A sterile glove was used as probe cover. Videos were recorded with sagittal and axial slices starting at the cranial end and finishing at the caudal end of the laminectomy window.

#### Postoperative

Patients underwent transcutaneous point-of-care USS of the injured spinal cord on days 1, 2, 3, 4, and 5 after surgery. A final spinal cord USS was performed on the same day as the postoperative MRI scan. Transcutaneous USS was performed with General Electric Venue R2 machine (General Electric, Boston, U.S.) using an abdominal convex probe (C1-5-RS, General Electric) at 1.4–5.7 MHz to reach the depth of the laminectomy. Videos were recorded with sagittal slices in a craniocaudal direction. Several technical challenges were overcome to allow postoperative transcutaneous USS imaging. Intraoperatively, a wide laminectomy was performed laterally to the facet joints. Objects that cast acoustic shadows near the midline, such as cross-links, were avoided and polyaxial screws were positioned as lateral as possible. The extradural subfascial drain tubing was placed away from the cord between facet joints and muscle. The wound was closed with nylon, avoiding staples, and covered with a narrow strip of MEPORE. An IOBAN drape was placed to provide a waterproof barrier to the USS jelly. After surgery, in the NICU, the patient was positioned left lateral. Care was taken to avoid applying pressure to the wound, which is known to cause cord compression and increase the ISP.^1,12^ The USS probe was placed paramedian, avoiding the midline, because the approximated skin, fascia, and muscle cast a strong acoustic shadow in the midline. We could thus obtain sagittal images of the cord, but no axial images. First, the USS probe was positioned in the sagittal plane paramedian, then the depth and gain were adjusted until the cord was visualized. Using this technique, the ultrasound penetrated to a maximum depth of 16 cm with a wide field of view allowing the CSF space dorsal to the cord to be visualized clearly in all cases, but often without a clear view of the CSF space ventral to the cord. We thus used the length of the spinal cord without the dorsal CSF as a measure of the degree of spinal cord compression.

### USS image analysis

USS videos were analyzed to determine the following parameters: (1) the presence or absence of spinal cord pulsatility, (2) maximum sagittal cord diameter (in millimeters) as well as the (3) sagittal degree of ventral, and (4) the dorsal CSF space effacement (in millimeters). Dorsal cord compression was defined as the length of spinal cord without CSF between the dorsal dura and spinal cord.

### ISP monitoring

The pressure probe was connected to a Codman ICP box linked to a Philips bedside monitor, which also received arterial line blood pressure and electrocardiogram lead II waveforms (Intellivue MX800; Philips, Guildford, United Kingdom). Data were sampled from the Philips bedside monitor using a laptop running LabChart PowerLab software (AD Instruments, Oxford, United Kingdom) up to August 2020, and thereafter the Intensive Care Monitor plus software (Cambridge Enterprise ICM+, Cambridge, United Kingdom). ISP is the same as intraparenchymal cord pressure at the injury site,^13^ which differs from CSF pressure above or below when the swollen spinal cord is compressed against the dura,^1^ thus compartmentalizing the intrathecal space.^14^ Mean ISP values were recorded in a 30-min window preceding the respective USS. ISP values were not available for intraoperative USS or for USS performed at the time of the postoperative MRI. Supplementary Data shows details of pressure probe insertion.

### ISP signal analysis

The raw ISP signal, sampled at 50–500 Hz, was cleaned of artifact. sAMP and sRAP were calculated from the ISP waveform as measures of compliance, as previously described for ICP.^4–8^ sAMP was defined as the mean peak-to-peak value (systolic ISP minus diastolic ISP), averaged every 10 sec. sRAP was defined as the correlation coefficient of 30 consecutive (x, y), where x = 10-sec averaged sAMP, and y = the corresponding mean ISP. When there is CSF around the injured spinal cord (good compensatory reserve), sAMP is low, and sRAP is 0 (i.e., no correlation between sAMP and mean ISP). When the spinal cord contacts the surrounding theca (compensatory reserve is exhausted), sAMP increases and sRAP approaches +1 (i.e., positive correlation between sAMP and mean ISP). By analogy with ICP,^9,15^ we hypothesized that, as compensatory reserve becomes increasingly impaired, ISP waveforms change shape from normal (class I: three peaks, P1 dominant), to probably pathological (class II: P2 dominant), to pathological (class III: rounded pulse, single peak). For each of the five patients who had mean ISP values spanning a wide range (0–50 mmHg), we classified 100 waveforms per mean ISP range (0–10, 10–20, 20–30, 30–40, 40–50) by inspection according to morphology.

### Statistics

Data in the figures are mean ± standard error. ΔISP = mean daily ISP minus ISP averaged over the first 3 h of recording (ΔISP = mean ISP—ISP_0–3h_). Similarly, ΔsAMP = mean sAMP—sAMP_0–3h_, and ΔsRAP = mean sRAP—sRAP_0–3h_. The effect of the following on ΔISP, ΔsAMP, and ΔsRAP was first determined by univariate analysis, and then, significant (*p* < 0.05) factors were examined by multivariate analysis using ANOVA: day after surgery, level of injury, admission AIS, time to surgery, decompression, age, and sex. Least-square (LS) means, calculated using ANOVA, are within-group means that are adjusted for other effects in the model.

## Results

### Patient characteristics

Details of the 79 patients included in the study are in Table 1. A total of 58 (73.4%) were male and 21 (26.6%) female, with their ages evenly spread between 19 and 75 years. The level of injury was cervical in 45 (57.0%), thoracic in 29 (36.7%), and lumbar-conus medullaris in 5 (6.3%) TSCIs. Admission AIS was grade A in 43 (54.4%), the rest were similarly distributed between AIS grades B in 17 (21.5%) and C in 19 (24.1%). Only 21 (26.6%) of the patients had surgery within 24 h of TSCI.

**Table 1.** Characteristics of the 79 Patients in the Study

Characteristic	Description	Value	Explanation
Age	Years	43 (19–75)	Mean (range)
Sex	Male	58 (73.4)	*n* (%)
	Female	21 (26.6)	
Level of injury	Cervical	45 (57.0)	*n* (%)
	Thoracic	29 (36.7)	
	Lumbar	5 (6.3)	
Admission AIS	A	43 (54.4)	*n* (%)
	B	17 (21.5)	
	C	19 (24.1)	
Time to surgery	0–24 h	21 (26.6)	*n* (%)
	24–48 h	36 (45.6)	
	48–72 h	22 (27.8)	
Decompression	No laminectomy	15 (19.0)	*n* (%)
	Laminectomy	64 (81.0)	
ISP monitoring (79 patients)	Hours	126.4 (17.4–181.8)	Mean (range)
USS monitoring (9 patients)	Number of USS	8.1 (3–18)	Mean (range)

ISP, intraspinal pressure; USS, ultrasound scanning.

### ISP monitoring

ISP monitoring, performed through laminotomy in about a fifth and laminectomy in four-fifths of 79 patients, produced a total of 9986 h of data, averaging about 5 days of ISP signal per patient (Table 1). A total of 9/79 (11.3%) patients had CSF leak from the wound drain or pressure probe exit site, successfully treated with reinforcing skin sutures. Wound infection occurred in 5/79 (6.3%) patients, evident >1 week after probe removal, who were successfully treated as follows: 1 had wound washout and revision, 3 had antibiotics, and 1 was managed conservatively with regular dressing changes. Asymptomatic noncompressive subfascial pseudomeningocoeles were diagnosed in 3/79 (3.8%) patients from the 2-week postoperative MRI. There were no incidences of spinal cord damage, hematoma, or meningitis.

### Spinal Monro–Kellie

We hypothesized that the Monro–Kellie doctrine, formulated to explain the relationship between intracranial volumes and ICP in TBI, also applies in TSCI. Three predictions of the spinal Monro–Kellie were tested as follows:

#### Prediction 1

**FIG. 1.**
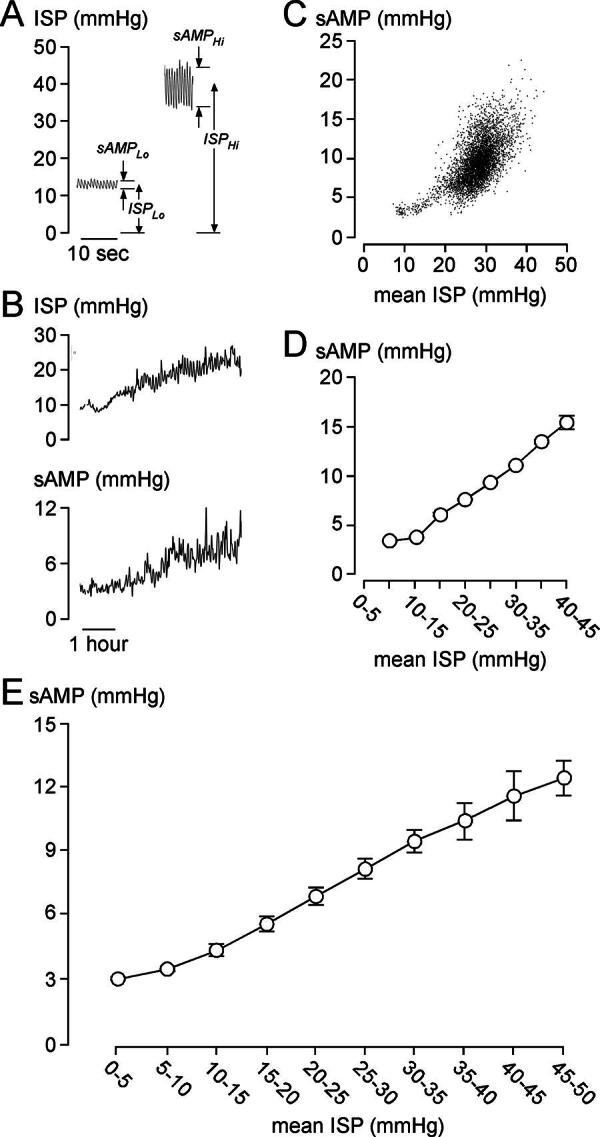
sAMP after TSCI. **(A)** Periods of low and high mean intraspinal pressure (ISP). Mean ISP (ISP_Lo_, ISP_Hi_) and corresponding ISP pulse amplitude (sAMP_Lo_, sAMP_Hi_). **(B)** Corresponding ISP (*top*), and sAMP (*bottom*) signals. **(C)** sAMP versus ISP and **(D)** sAMP versus grouped ISP, averaged every minute for entire 86.5 h of monitoring. **A–D**: 55-year-old, male, T7 level of injury, AIS grade A. **(E)** sAMP versus ISP for all 79 patients. **D–E**: Mean ± standard error.

*As ISP increases, sAMP is initially unchanged, then increases.* Figure 1 shows examples of low and high mean ISP and their relation to sAMP as well as a representative 6-h window of the ISP signal with the corresponding sAMP signal recorded from a 55-year-old man with AIS grade A TSCI at T7. When ISP is 10–15 mmHg, sAMP appears constant, then sAMP rapidly increases as ISP increases >15 mmHg. Plot of sAMP versus ISP, averaged every 60 sec, and the grouped data of the entire ISP signal of this patient, confirms this relationship. sAMP versus ISP plotted using data from the ISP signals of all 79 patients also supports the relationship predicted by the spinal Monro–Kellie and indicates that, on average, the compensatory reserve begins to reduce as ISP rises above 10 mmHg. Unlike ICP, no critical ISP (beyond which sAMP decreases with increasing ISP) and no Lundberg A-waves were seen in any of the patients.^5,6^

#### Prediction 2

**FIG. 2.**
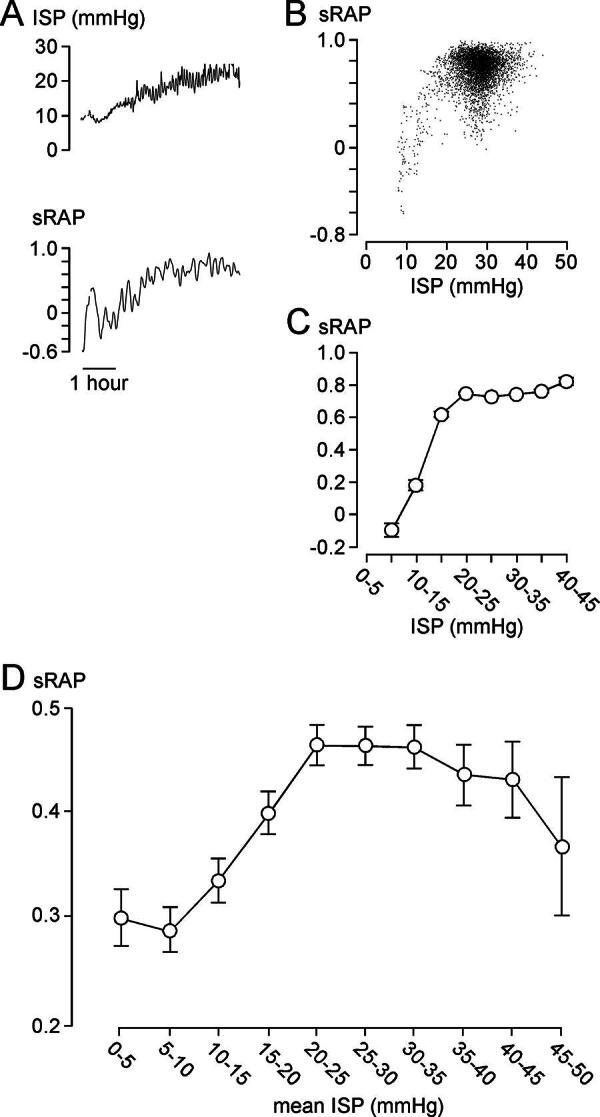
sRAP after traumatic spinal cord injury (TSCI). **(A)** Corresponding ISP (*top*), and sAMP (*bottom*) signals. **(B)** sRAP versus ISP and **(C)** sRAP versus grouped ISP, averaged every minute for entire 86.5 h of monitoring. **A–C**: 55-year-old, male, T7 level of injury, AIS grade A. **(D)** sRAP versus ISP for all 79 patients. **C–D**: mean ± standard error.

*As ISP increases, sRAP is initially unchanged, then increases.* Figure 2 shows the same 6-h ISP signal as in Figure 1, with the corresponding sRAP signal. sRAP rapidly increases as ISP increases >10 mmHg. Plot of sRAP versus ISP, averaged every 60 sec, and the grouped data of the entire 86.5-h-long ISP signal of this patient, confirms this relationship. sRAP versus ISP plotted using data from the ISP signals of all 79 patients also supports the relationship predicted by the spinal Monro–Kellie and indicates that, on average, the compensatory reserve begins to reduce as ISP rises above 10 mmHg. Again, no critical ISP (beyond which sRAP becomes negative with increasing ISP) was found in any of the patients.^5,6^

#### Prediction 3

**FIG. 3.**
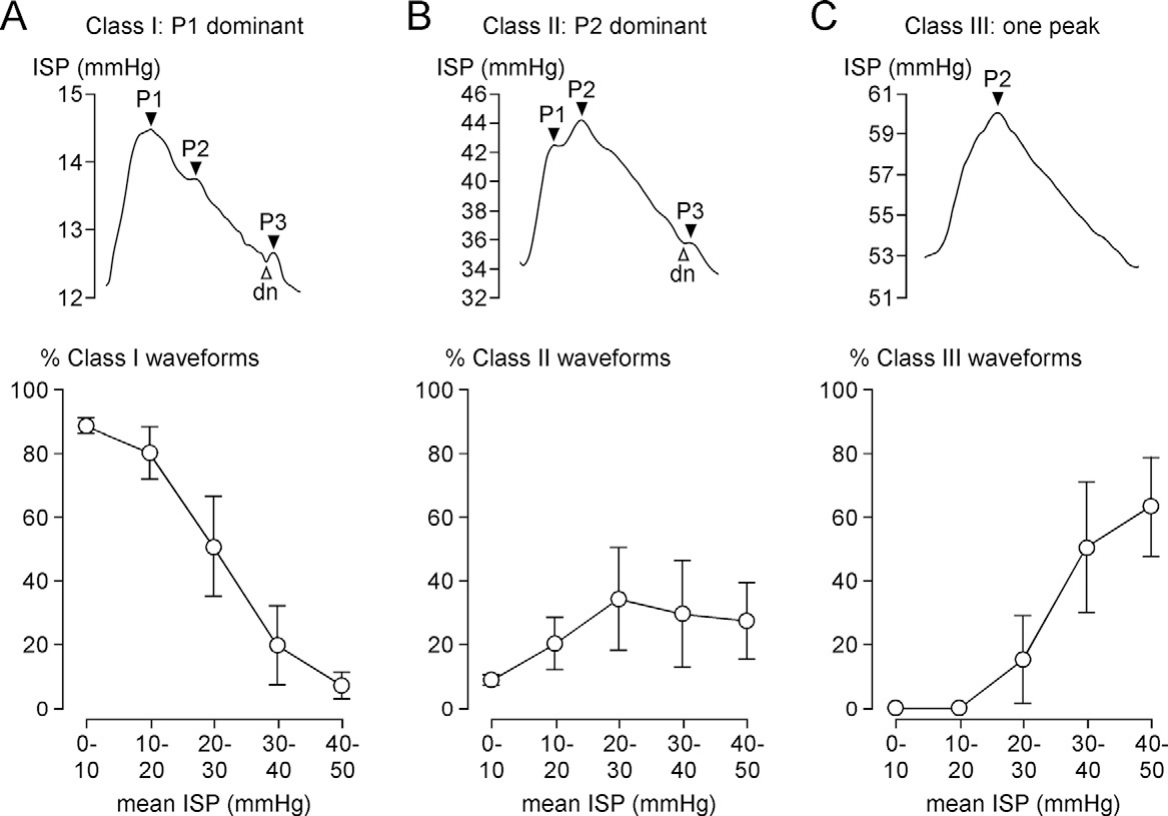
Morphology of ISP waveform. **(A)** Class I waveform: P1 dominant peak (*top*). % Class I waveforms versus mean ISP (*bottom*). **(B)** Class II waveform: P2 dominant peak (*top*). % Class II waveforms versus mean ISP (*bottom*). **(C)** Class III waveform: one peak only (*top*). % Class III waveforms versus mean ISP (*bottom*). One hundred waveforms at each mean ISP range were classified from each of five TSCI patients. % artifact waveforms (ISP mmHg): 2.8 ± 1.5 (0–10), 0.0 ± 0.0 (10–20), 0.0 ± 0.0 (20–30), 0.4 ± 0.4 (30–40), 2.4 ± 2.4 (40–50). Mean ± standard error. P1, P2, P3 (*black arrowheads*), dn, dicrotic notch (*white arrowhead*).

*As ISP increases, the ISP waveform changes shape indicating reduced compliance.* Examples of the three classes of ISP waveforms (Class I: three peaks, P1 dominant; Class II: three peaks, P2 dominant; Class III: one peak) are shown in Figure 3. As mean ISP increases, the percentage of Class I waveforms decreases, and Class II and III waveforms become more frequent, with Class III becoming the predominant waveform at mean ISP >30 mmHg, thus supporting the prediction of the spinal Monro–Kellie.

**FIG. 4.**
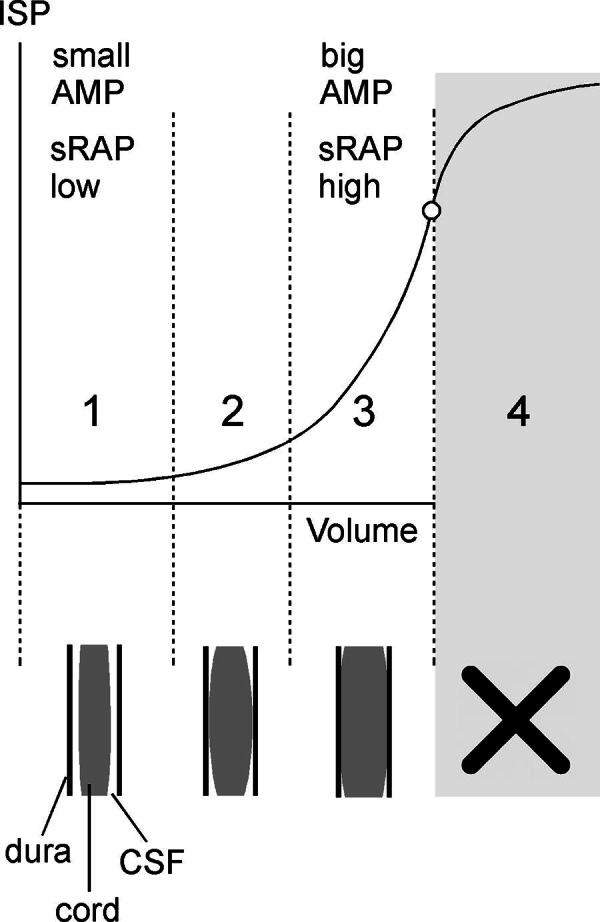
Pressure–volume curve. Schematic of pressure–volume curve for spinal cord. Phase 1: Compensation (cerebrospinal fluid [CSF] around cord; ISP, sAMP, sRAP all low), Phase 2: Early decompensation (cord contacts dura; ISP, sAMP, sRAP all begin to rise), Phase 3: Exponential decompensation (cord compressed against dura; ISP, sAMP, sRAP all high), Phase 4: (microvascular collapse; beyond a critical pressure sAMP decreases, sRAP negative) seen in traumatic brain injury (TBI) brain does not apply to TSCI.

The data from Figures 1–3 suggest that, after TSCI, the injured cord has the spinal pressure–volume curve, shown in Figure 4. In Phase 1 (compensated), there is CSF around the injured cord and thus ISP, sAMP, and sRAP are low. Phase 2 (early decompensation) occurs when the swollen, injured cord begins to contact the theca, and thus, ISP, sAMP, and sRAP begin to increase. In Phase 3 (exponential decompensation), the swollen cord becomes compressed against theca and thus a small increase in volume causes a large increase in ISP, sAMP, and sRAP. The cord does not have Phase 4, found in the brain, when microvascular collapse causes AMP to decrease and RAP to become negative as ICP increases beyond a critical value.

### Changes in ISP, sAMP, and sRAP after surgery

**FIG. 5.**
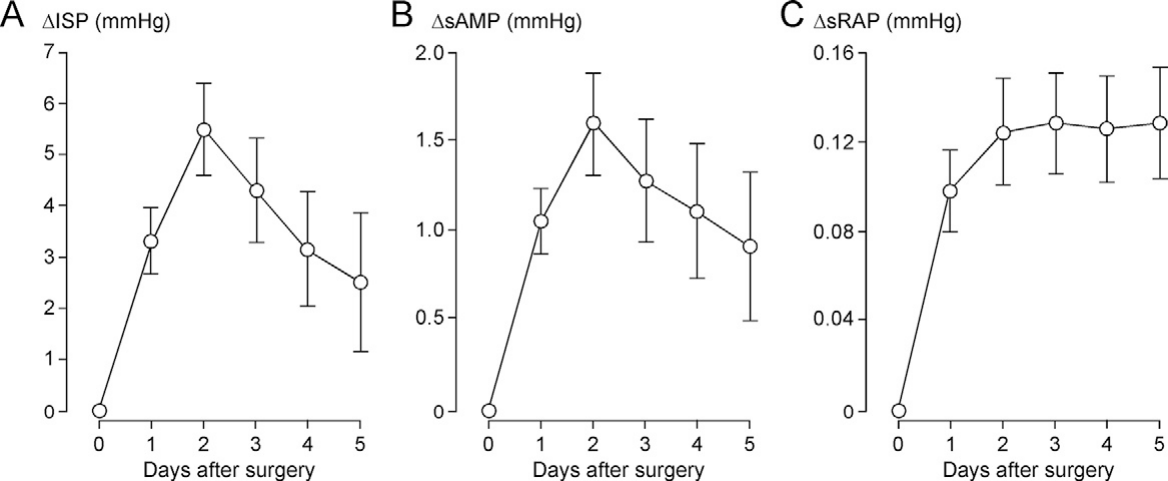
Changes in ISP, sAMP, and sRAP after surgery. **(A)** Mean daily ISP minus ISP averaged over the first 3 h postoperatively versus days after surgery. **(B)** Mean daily sAMP minus sAMP averaged over the first 3 h postoperatively versus days after surgery. **(C)** Mean daily sRAP minus sRAP averaged over the first 3 h postoperatively versus days after surgery. Mean ± standard error, 79 patients.

For each of the 79 patients, we computed ΔISP, ΔsAMP, and ΔsRAP as described in the Materials and Methods section. Positive ΔISP indicates increased cord swelling, whereas positive ΔsAMP and ΔsRAP indicate reduced compensatory reserve, compared with immediately after surgery. Figure 5 shows that over the first 5 days postoperatively, ISP, sAMP, and sRAP all increase, with ISP and sAMP peaking at 48 h postoperatively. Supplementary Figure S1 shows examples of increasing ISP in the first day after surgery as well as the baseline ISP of all 79 patients averaged at 0–3 h postoperatively. In univariate analysis, the thoracic level of injury, lower AIS grade on admission, younger age, and male sex were significant risk factors for delayed rise in ISP, but in multivariate analysis, only thoracic level of injury and lower AIS grade on admission remained significant risk factors for delayed rise in ISP (Table 2). Supplementary Figure S2 shows the ΔISP for 5 days after surgery in cervical versus thoracic injuries, and in patients with motor complete (AIS grades A and B) versus motor incomplete (AIS grade C) TSCI. In univariate analysis, thoracic level of injury, lower AIS grade on admission, no laminectomy, and male sex were significant risk factors for delayed rise in sAMP, but in multivariate analysis, only the thoracic level of injury and no laminectomy remained significant risk factors for delayed rise in sAMP (Table 3). Supplementary Figure S3 shows the baseline sAMP of all 79 patients averaged at 0–3 h postoperatively as well as the ΔsAMP for 5 days after surgery in cervical versus thoracic injuries, and in patients who had laminectomy versus no laminectomy. In univariate analysis, thoracic level of injury, lower AIS grade on admission, and delayed surgery (>24 h from TSCI) were significant risk factors for delayed rise in sRAP, but in multivariate analysis, only thoracic level of injury and delayed surgery (>24 h from TSCI) remained significant risk factors for delayed rise in sRAP (Table 4). Supplementary Figure S4 shows the baseline sRAP of all 79 patients averaged at 0–3 h postoperatively as well as the ΔsRAP for 5 days after surgery in cervical versus thoracic injuries, and in patients who had surgery ≤24 h versus >24 h after TSCI. Together, these findings suggest that, after surgery, severe thoracic injuries swell more, and that, in thoracic injuries without laminectomy that have delayed surgery, compensatory reserve worsens more.

**Table 2.** Analysis of Factors Associated with Postoperative Rise in Intraspinal Pressure

Factors	LS means		*F* statistic	*p* value
Univariate				
Day after surgery	1, 2, 3, 4, 5		1.4	ns
Level of injury	Cervical	2.7	15.1	<0.0005
	Thoracic	5.8		
Admission AIS	Motor complete	4.6	12.3	<0.001
	Motor incomplete	1.4		
Time to surgery	≤24 h		3.3	ns
	>24 h			
Decompression	No laminectomy		1.37	ns
	Laminectomy			
Age	<60 years	4.3	4.4	<0.05
	≥60 years	2.3		
Sex	Female	2.1	6.7	<0.01
	Male	4.4		
Multivariate				
Level of injury	Cervical	1.7	11.1	<0.005
	Thoracic	4.7		
Admission AIS	Motor complete	4.3	5.0	<0.05
	Motor incomplete	2.1		
Age	<60 years		0.8	ns
	≥60 years			
Sex	Female		0.1	ns
	Male			

LS, least squares; ns, not significant.

**Table 3.** Analysis of Factors Associated with Postoperative Rise in Pulse Amplitude

Factors	LS means (mmHg)		*F* statistic	*p* value
Univariate				
Day after surgery	1, 2, 3, 4, 5		0.7	ns
Level of injury	Cervical	0.5	54.7	<0.0001
	Thoracic	2.4		
Admission AIS	Motor complete	1.5	17.2	<0.0001
	Motor incomplete	0.3		
Time to surgery	≤24 h		3.3	ns
	>24 h			
Decompression	No laminectomy	2.0	5.9	<0.05
	Laminectomy	1.1		
Age	<60 years		2.8	ns
	≥60 years			
Sex	Female	0.7	6.5	<0.05
	Male	1.4		
Multivariate				
Level of injury	Cervical	0.7	46.9	<0.0001
	Thoracic	2.6		
Admission AIS	Motor complete	1.5	3.3	ns
	Motor incomplete	0.3		
Decompression	No laminectomy	2.3	14.7	<0.0005
	Laminectomy	1.0		
Sex	Female	0.7	0.8	ns
	Male	1.4		

LS, least squares; ns, not significant.

**Table 4.** Analysis of Factors Associated with Postoperative Rise in sRAP

Factors	LS means		*F* statistic	*p* value
Univariate				
Day after surgery	1, 2, 3, 4, 5		0.2	ns
Level of injury	Cervical	0.09	13.0	<0.0005
	Thoracic	0.17		
Admission AIS	Motor complete	0.13	5.3	<0.05
	Motor incomplete	0.08		
Time to surgery	≤24 h	0.07	10.3	<0.005
	>24 h	0.14		
Decompression	No laminectomy		1.3	ns
	Laminectomy			
Age	<60 years		0.2	ns
	≥60 years			
Sex	Female		0.0	ns
	Male			
Multivariate				
Level of injury	Cervical	0.08	7.4	<0.01
	Thoracic	0.14		
Admission AIS	Motor complete		1.1	ns
	Motor incomplete			
Time to surgery	≤24 h	0.08	6.4	<0.05
	>24 h	0.14		

LS, least squares; ns, not significant.

### Serial USS

**FIG. 6.**
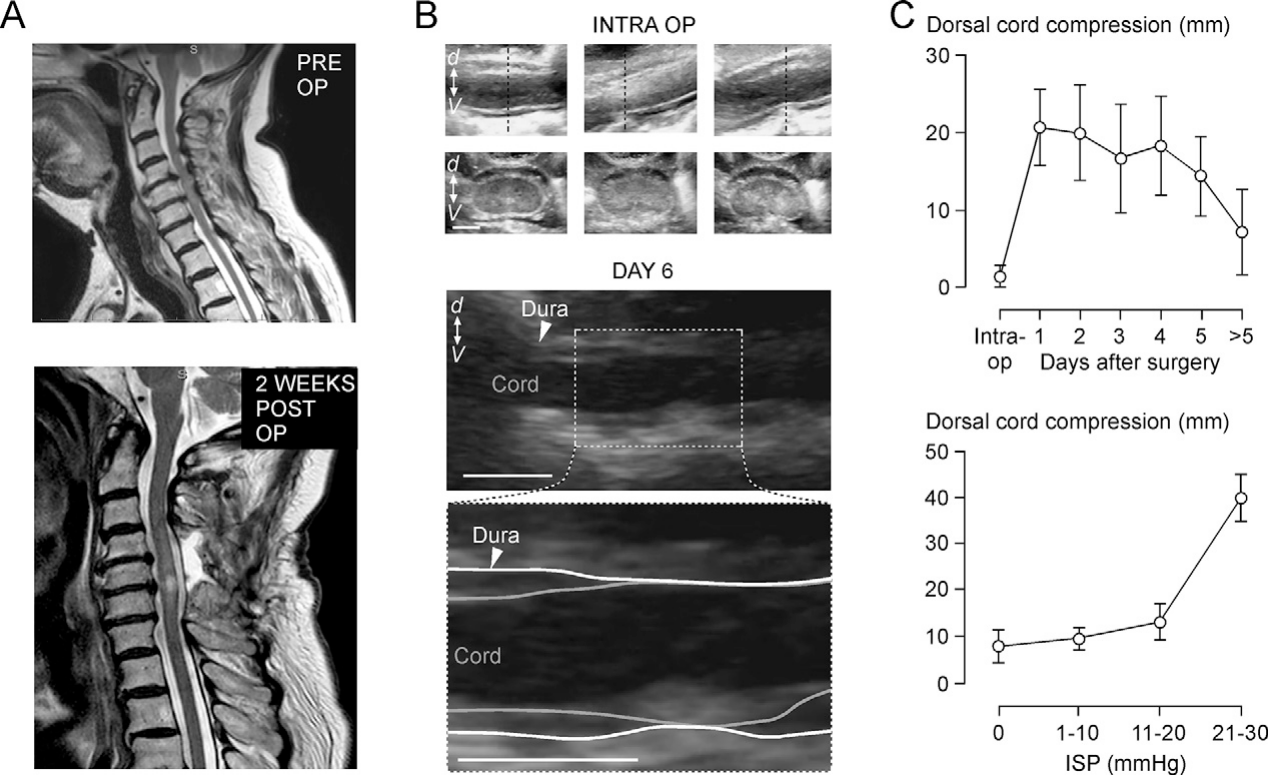
Serial ultrasound scans of injured spinal cord. **(A)** Preoperative (*top*) and 2-week postoperative (*bottom*) magnetic resonance imaging (MRI) of a TSCI patient (75-year-old, female, C6 level of injury, AIS grade A). **(B)** Ultrasound scanning (USS) obtained intraoperatively after laminectomy (*top*) and on day 6 after surgery (*bottom*). Intraoperative longitudinal (*top*) and axial through dotted line (*bottom*) USS at C4 (*left*), C5 (*middle*), and C6 (*bottom*). Day 6 longitudinal USS with magnified view of boxed area below. d↔v, dorsal–ventral axis. Bar = 1 cm. **(C)** Plots of dorsal cord compression versus days after surgery (*top*) and dorsal cord compression versus ISP. Mean ± standard error.

We then used USS, looking for delayed cord swelling. Serial USS was obtained in nine patients, with one intraoperative and seven postoperative scans per patient on average (Table 1). Intraoperative USS performed after decompression including laminectomy revealed adequate cord decompression with CSF evident dorsal and ventral to the spinal cord in axial and sagittal views in all nine patients (Fig. 6). Dorsal cord compression increased from no compression intraoperatively to about 2 cm compression at postoperative day 1 and took >5 days for the injured cord to decompress (Fig. 6). Representative USS taken intraoperatively (no compression), at 4 days (thecal compression), and at 2 weeks (no compression) after surgery from the patient in Figure 6 is shown as Supplementary Videos S1, S2, and S3, respectively. Figure 6 also shows that the extent of dorsal cord compression thus determined using USS positively correlates with the ISP averaged during the scanning period. Together, the USS findings suggest that the injured cord swells and may become compressed against the theca in the days after surgery.

## Discussion

Our key finding is that the Monro–Kellie doctrine, which was developed to explain the pressure–volume relationship for the brain, also applies for the spinal cord. We also showed that, after TSCI, cord swelling is dynamic and increases within the first 5 days after surgery.

### Spinal Monro–Kellie doctrine

The evidence supporting the notion of spinal Monro–Kellie doctrine is strong. Using three measures of compensatory reserve from ISP signal analysis (sAMP, sRAP, morphology of ISP waveform), we showed that, as the cord swells, compensatory reserve becomes exhausted. The relationships between sAMP, sRAP, and morphology of ISP versus mean ISP in TSCI are remarkably similar to the corresponding relationships between AMP, RAP, and the morphology of ICP versus mean ICP in TBI.^4–9^

There are two notable differences between TBI and TSCI. First, is the lack of ISP plateau waves, analogous to the ICP plateau waves also known as Lundberg A waves. In TBI, the ICP plateau waves are sudden elevations in ICP typically >50 mmHg for >5 min.^16^ They are a hemodynamic phenomenon arising from cerebral vasodilatation when the CPP drops in patients with intact autoregulation but exhausted compensatory reserve.^17^ The second difference between TBI and TSCI is the lack of a critical ISP beyond which sRAP <0, analogous to the critical ICP in TBI. In TBI, when the ICP exceeds this critical ICP level, PRx becomes exhausted, AMP becomes inversely related to ICP, and thus RAP is –1.^7^ At this point cerebral ischemia-induced irreversible brain damage will likely occur with ultimate brain herniation. These hemodynamic differences between the brain and spinal cord may be related to the reduced blood supply of the spinal cord compared with the brain^18–20^ and the fact that the spinal canal is not a completely “closed box” and thus the injured cord initially swells and is compressed against the surrounding theca, but then the cord swelling may dissipate above and below the injury site thus preventing the terminal loss of pressure reactivity.

The effect of MAP on compliance has not been studied here but is of interest given the guideline to maintain MAP for a week after TSCI at 85–95 mmHg. From our earlier work,^1,21^ when autoregulation is impaired (sPRx >0), increasing MAP increases spinal cord blood volume thus increasing spinal cord swelling, in turn causing a decrease in compliance. Conversely, when autoregulation is intact (sPRx ≤0), increasing MAP decreases spinal cord blood volume thus reducing spinal cord swelling, in turn causing an increase in compliance.

### Delayed spinal cord swelling

Our findings of increased spinal cord swelling and loss of compensatory reserve after surgery, shown here as rise in ISP, sAMP, and sRAP, are supported by studies of serial MRI after TSCI showing increased cord edema over the few days after TSCI.^22–24^ Delayed spinal cord edema after TSCI has also been described in animals, and occurs between 72 h and 5 days after injury depending on model species, injury severity, and injury location.^25^

In our study, four factors were associated with increased spinal cord swelling and/or loss of compliance after surgery in multivariate analysis as follows:

1. Thoracic level of injury. The reasons may be twofold. First, compared with the cervical cord, the thoracic cord is less well perfused with a watershed blood supply.^18^ Second, the force required to damage the thoracic spine is greater than the cervical spine because the thoracic spine is supported by ribs and sternum and thus thoracic AIS grade A TSCI is generally more severe than cervical AIS grade A TSCI.^26^
2. No laminectomy. This is supported by the observation that laminectomy improves cord decompression, that is, increases the likelihood of CSF signal around the injured spinal cord on postoperative MRI.^27^
3. Delayed surgery (>24 h from TSCI). This finding is supported by a substantial body of indirect evidence that early decompression improves neurological outcome after TSCI based on the STASCIS trial^28^ and pooled analysis of individual patient data derived from four independent, prospective, multicenter data sources.^29^
4. More severe TSCI. More severe tissue damage is expected to incur more cytotoxic edema and more severe vascular damage with more vasogenic edema.^25^

It is interesting to note that delayed spinal cord swelling still occurred, although to a lesser extent, in cervical injuries, after laminectomy, and despite early surgery. This observation raises the possibility that the theca is a key contributor to spinal cord compression in these patients and thus duroplasty may reduce cord compression and improve outcome.^30^ Our finding that age was not significantly linked to delayed spinal cord swelling in multivariable analysis suggests that the central cord syndrome is not immune to delayed cord swelling.

### Ultrasound scanning

Spinal cord swelling was further assessed using intraoperative and postoperative USS. Intraoperative USS is becoming increasingly popular among spinal surgeons to show adequate cord decompression during surgery not only in TSCI but also in cervical myelopathy. USS is easy to use, provides clear images, excellent operator-dependent reproducibility, is a nonionizing imaging modality, and provides the potential for perfusion assessment and neuromodulation.^31–33^ Postoperative transcutaneous USS to visualize the spinal cord has not been widely used, but there are reports of such imaging after laminectomy for spinal cord tumors^34^ and after cervical laminoplasty to assess adequate decompression, cord pulsation, and development of epidural hematoma.^35^ In our hands, USS of the cord was possible following a learning curve, and allowed serial imaging of the injured cord without having to transfer the patients from the NICU to the MRI unit. Our USS measure of compression, dorsal cord compromise, positively correlated with mean ISP measured at the time of scanning thus validating this USS measure of compression. The key finding of the USS study is that cord compression may develop after surgery in patients who had intraoperative USS confirmation of satisfactory cord decompression. ISP monitoring for many days after surgery is required to monitor the development of delayed cord edema.

### Clinical implications

The spinal Monro–Kellie doctrine, proposed here, has important clinical implications for the management of TSCI. As the injured cord swells, the surrounding compensatory reserve volume becomes exhausted thus causing a rise in ISP, reduction of spinal cord blood flow, ischemic secondary injury, and ultimately, spinal cord infarction at the injury site. Early spinal cord decompression after TSCI therefore makes sense, akin to decompressive craniectomy in TBI. In TSCI, effective decompression would require a durotomy, in addition to bony decompression. The effect of performing duroplasty in acute, severe cervical TSCI in addition to bony decompression is currently investigated in the DISCUS randomized controlled trial.^30^ Our findings suggest that measuring ISP by transducing from a lumbar catheter^36^ is accurate only when there is compensatory reserve volume around the injury site but becomes inaccurate when the compensatory reserve volume is exhausted. Thus, ISP can only be accurately determined from the injury site, as we previously showed.^37^ This also means that draining the lumbar CSF would only effectively increase SCPP if there is compensatory reserve volume surrounding the injured cord but, when the compensatory reserve volume is exhausted, draining lumbar CSF will not alter SCPP and may even be detrimental by causing downward herniation of the swollen, injured spinal cord.^37^ Measuring ISP from the injury site has not been widely adopted likely due to the reluctance of spinal surgeons to breach the theca after TSCI risking CSF leak, probe-related spinal cord damage, and meningitis. A review of 42 patients who had ISP monitoring reported no serious complications.^12^

## Conclusions

We described a novel concept, the spinal Monro–Kellie doctrine, that explains the pressure–volume relationship of the spinal cord. This allowed the concepts of sAMP and sRAP to be defined for quantifying compensatory reserve in TSCI. The spinal Monro–Kellie doctrine is analogous to its brain counterpart but has notable differences. We also presented evidence of delayed spinal cord swelling after surgery that may render the practice of using USS, to confirm adequate cord decompression during surgery, falsely reassuring.

## Transparency, Rigor, and Reproducibility Statement

The ISCoPE study was preregistered at www.clinicaltrials.gov and www.ichgcp.net as NCT02721615. Because this is the first study of its kind in this cohort, as many cases as possible from the ISCoPE database were analyzed; therefore, there is no formal sample size calculation. Seventy-nine patients had intraspinal pressure data amenable to analysis, including nine patients for serial ultrasound scans. The intraspinal pressure signals were analyzed using techniques already validated for intracranial pressure signal analysis, including multivariate analysis to correct for multiple comparisons. There are no established standard techniques for analyzing the ultrasound images of the spinal cord. Data collection and analyses were performed by investigators who were aware of relevant characteristics of the participants. No replication or external validation studies have been performed or are planned/ongoing at this time. The raw data used to conduct the analyses presented in this study are not available in a public repository. Data may be available by e-mailing the corresponding author, Dr. Samira Saadoun. The authors agree to provide the full content of the article on request.

## Abbreviations Used

AIS: American spinal injury association Impairment Scale
AMP: pulse AMPlitude
CSF: Cerebro Spinal Fluid
CPP: Cerebral Perfusion Pressure
ICP: Intra Cranial Pressure
ISCoPE: Injured Spinal Cord Pressure Evaluation study
ISP: Intra Spinal Pressure
MAP: Mean Arterial Pressure
NICU: Neuro Intensive Care Unit
RAP: correlation coefficient (R) between AMP and mean ISP
SCPP: Spinal Cord Perfusion Pressure
TBI: Traumatic Brain Injury
TSCI: Traumatic Spinal Cord Injury
USS: Ultra Sound Scan
