# Supplementary material for: Evolution of Spinal Cord Swelling in Acute Traumatic Spinal Cord Injury
Source: Neurotrauma Rep. 2025 Feb 12;6(1):158–70. doi: 10.1089/neur.2025.0005 (PMC11931111; doi:10.1089/neur.2025.0005)

**SUPPLEMENTARY METHODS: INSERTION OF ISP PROBE**

After reducing and fixing the spinal fracture and inserting metalwork to stabilize the

spine, a 14-gauge introducer is used to tunnel the Codman probe through the skin into the wound. We use a 21-gauge needle bent at 90° to perforate the dura one level below the injury. To monitor ISP, the Codman probe is zeroed in saline then advanced through the dural hole

until the probe tip is at the site of maximal spinal cord swelling judged to be the centre of cord contusion/oedema from the pre-operative MRI and confirmed on intraoperative USS. The probe is curled and secured to the skin with silk sutures to avoid kinking and migration. A tightening stitch is placed around the probe exit site to prevent CSF leak. Tunnelling the probe through fascia and paraspinal muscle reduces the risk of CSF leak around the probe skin exit site. The probe is connected to a Codman ICP box then ICM+ via a Philips Intellivue ICU bedside monitor. The skin is closed with a locking nylon suture, Opsite® spray is applied and Ioban® drape is applied to minimize the risk of CSF leak through the wound and the risk of infection. A subfascial wound drain is inserted and placed on gravity, left in situ for one week. Probe tip positioning is confirmed on a postoperative CT scan when assessing fixation metalwork to ensure probe migration has not occurred. In these patients with blunt injuries, the dura and arachnoid are intact. Placing the pressure probe above or below the arachnoid makes no difference to the ISP signal. The probe is inserted and advanced parallel to the dura and is situated extramedullary to eliminate the risk of spinal cord damage. The steps involved in inserting the probe and recording the ISP signal are summarised below, which is based on Figure 1 from *Critical Care Medicine* 2014;42:646-55.


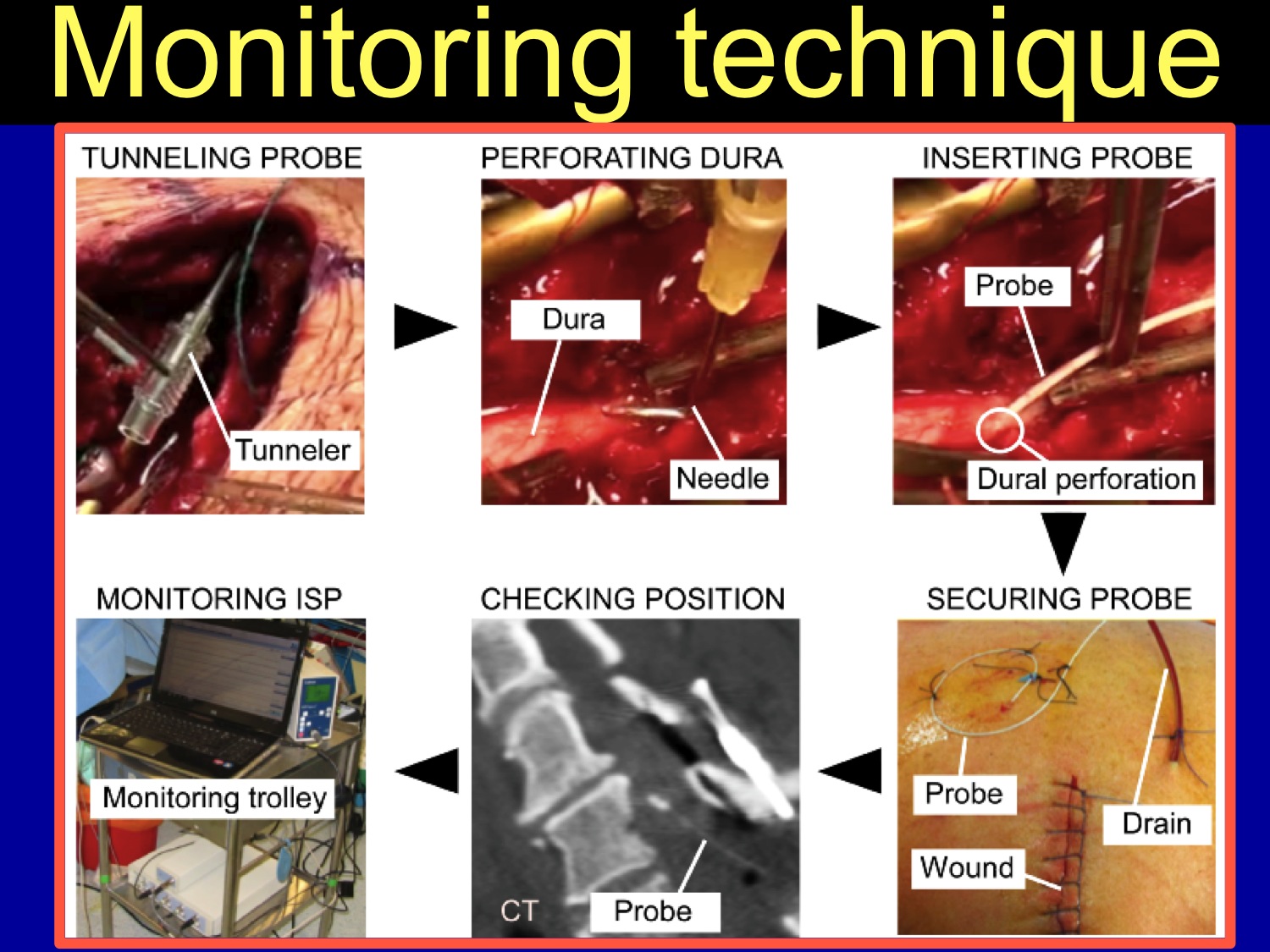

Supplement: Supplementary Data [file neur.2025.0005_supplementary_data.docx]
