# Supplementary material for: Evolution of Spinal Cord Swelling in Acute Traumatic Spinal Cord Injury
Source: Neurotrauma Rep. 2025 Feb 12;6(1):158–70. doi: 10.1089/neur.2025.0005 (PMC11931111; doi:10.1089/neur.2025.0005)
Supplement: Supplementary Figure S1 [file neur.2025.0005_supplementary_figure_s1.pdf]

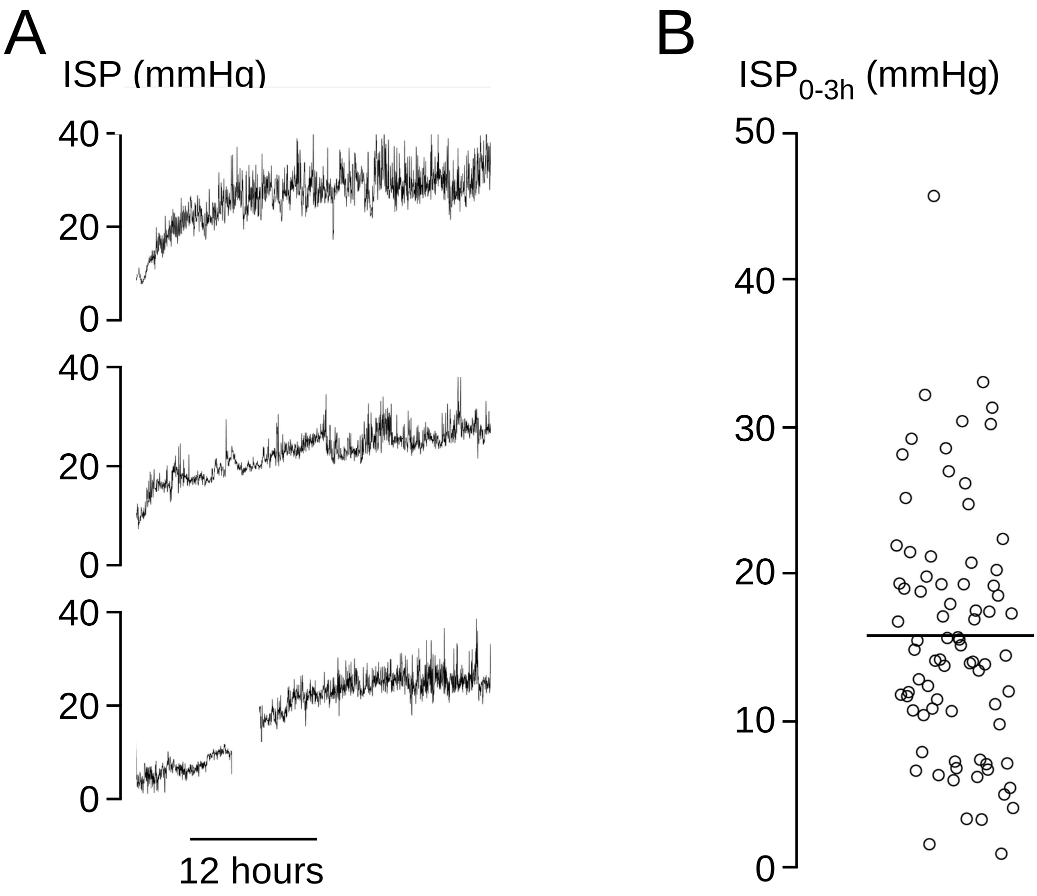

Delayed rise in ISP after TSCI. A. ISP traces from three TSCI patients. (top) 55-year-old, male, T7 level of injury, AIS grade A (middle) 56-year-old, female, L1 level of injury, AIS grade C (bottom) 58-year-old, male, T8 level of injury, AIS grade B. **B.** ISP values averaged over the first 3 h after surgery. Each dot is a patient, 79 patients, line is mean.
