# Supplementary material for: Evolution of Spinal Cord Swelling in Acute Traumatic Spinal Cord Injury
Source: Neurotrauma Rep. 2025 Feb 12;6(1):158–70. doi: 10.1089/neur.2025.0005 (PMC11931111; doi:10.1089/neur.2025.0005)
Supplement: Supplementary Figure S2 [file neur.2025.0005_supplementary_figure_s2.pdf]

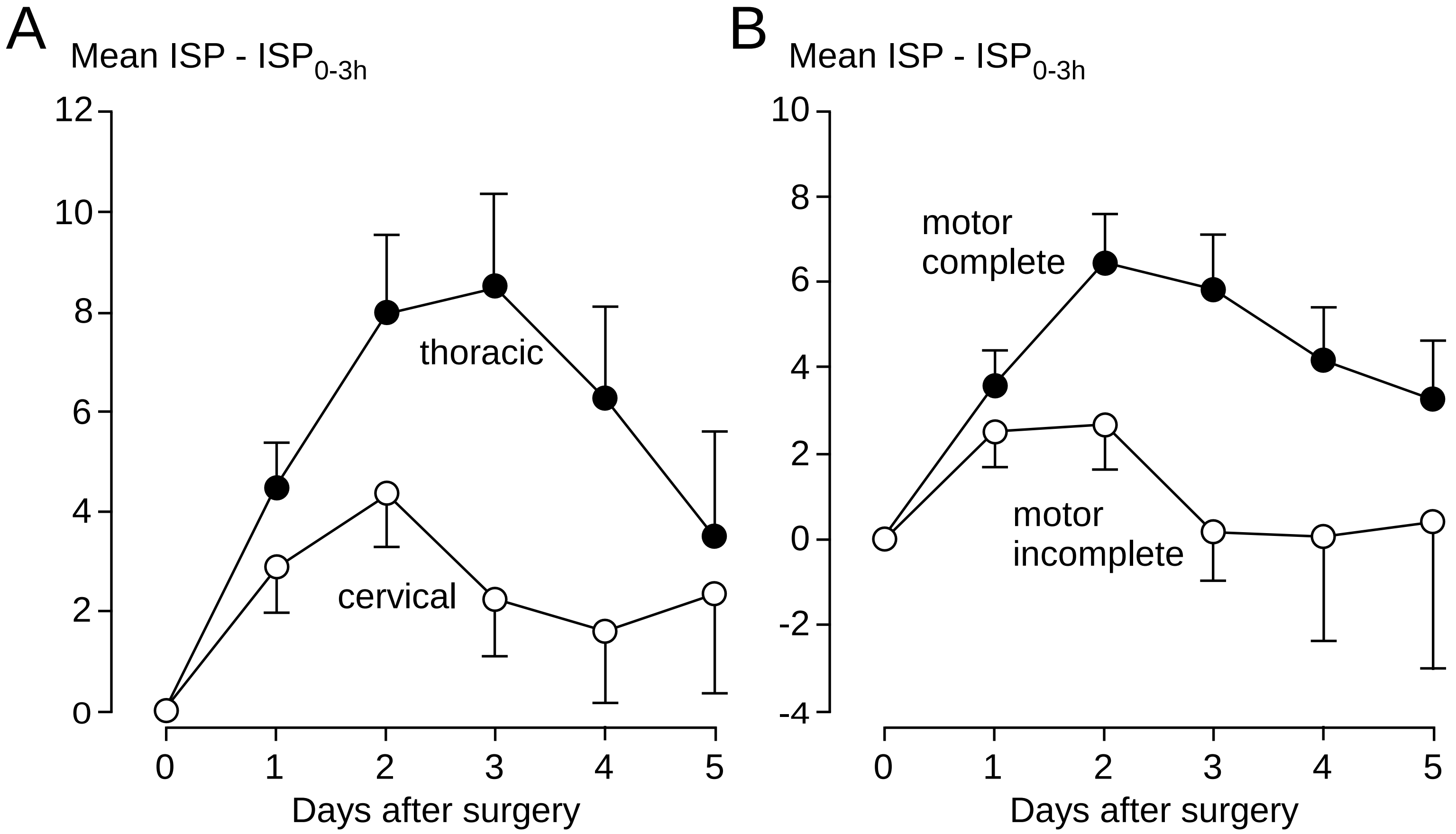

Factors affecting delayed ISP rise after TSCI. A. 45 patients with cervical TSCI, 29 patients with thoracic TSCI. **B.** 60 patients with motor complete TSCI, 19 patients with motor incomplete TSCI. Mean ± standard error.
