# Supplementary material for: Evolution of Spinal Cord Swelling in Acute Traumatic Spinal Cord Injury
Source: Neurotrauma Rep. 2025 Feb 12;6(1):158–70. doi: 10.1089/neur.2025.0005 (PMC11931111; doi:10.1089/neur.2025.0005)
Supplement: Supplementary Figure S3 [file neur.2025.0005_supplementary_figure_s3.pdf]

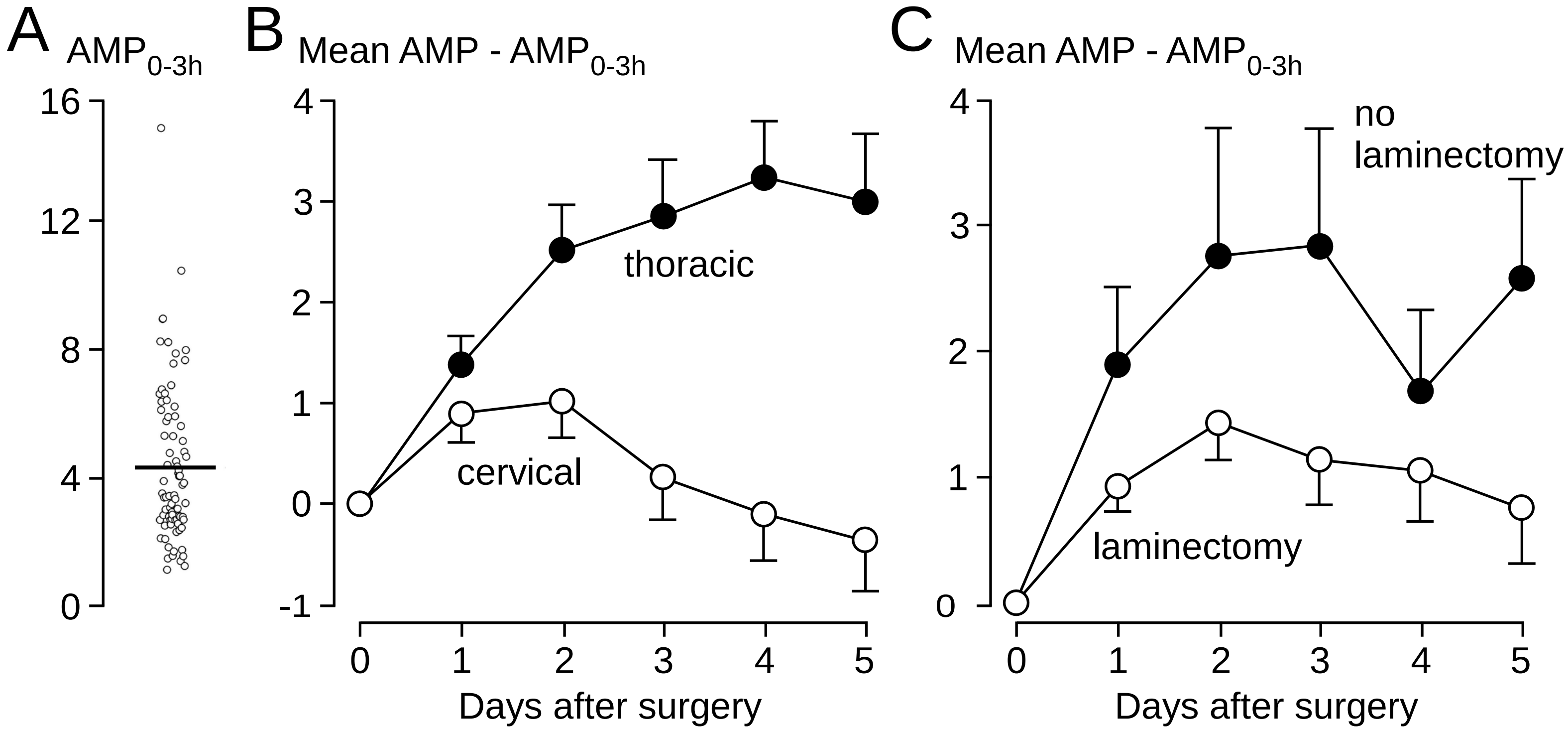

Factors affecting delayed loss of compensatory reserve (increased sAMP) after TSCI.

A. AMP values averaged over the first 3 h after surgery. Each dot is a patient, 79 patients, line is mean. Mean daily AMP minus AMP averaged over the first 3 h postoperatively vs. days after surgery for **B.** 45 patients with cervical TSCI, 29 patients with thoracic TSCI, and **C.** 11 patients without laminectomy, 68 patients with laminectomy. Mean ± standard error.
