# Supplementary material for: Evolution of Spinal Cord Swelling in Acute Traumatic Spinal Cord Injury
Source: Neurotrauma Rep. 2025 Feb 12;6(1):158–70. doi: 10.1089/neur.2025.0005 (PMC11931111; doi:10.1089/neur.2025.0005)
Supplement: Supplementary Figure S4 [file neur.2025.0005_supplementary_figure_s4.pdf]

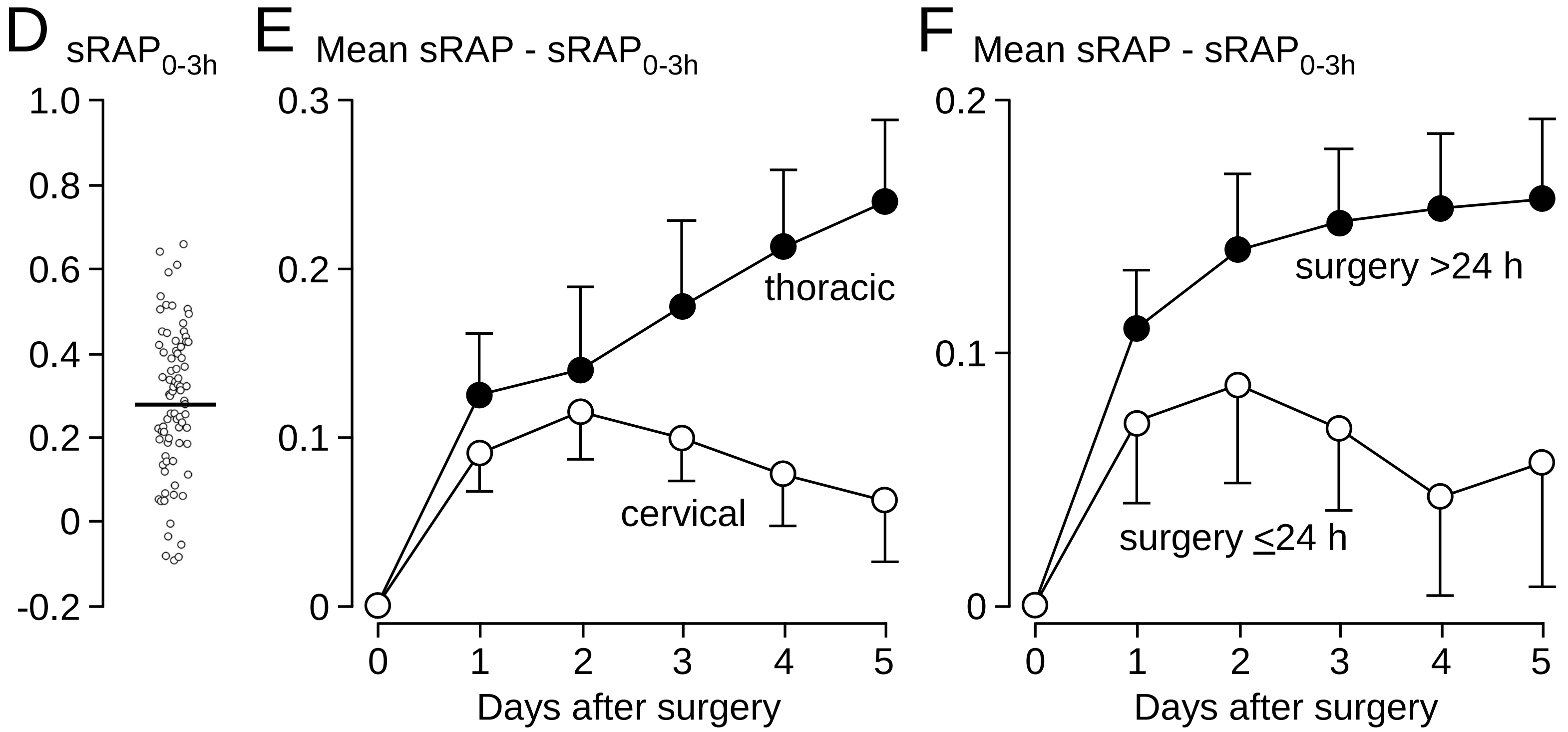

Factors affecting delayed loss of compensatory reserve (increased sRAP) after TSCI. **A.** sRAP values averaged over the first 3 h after surgery. Each dot is a patient, 79 patients, line is mean. Mean daily sRAP minus sRAP averaged over the first 3 h postoperatively vs. days after surgery for **B.** 45 patients with cervical TSCI, 29 patients with thoracic TSCI, and **C.** 21 patients had surgery within 24 h of TSCI, 58 patients 24 - 72 h of TSCI. Mean ± standard error.
